# Lessons from leadership transition of an AMR telementoring program to sustain laboratory capacity building in Ethiopia

**DOI:** 10.1017/ash.2023.405

**Published:** 2023-09-29

**Authors:** Martin Evans, Ana Da Costa, Kieran Hartsough, Jacqueline Safstrom, Carolyn Herzig, Rajiha Abubeker, Gebrie Alebachew, Surafel Fentaw Dinku, Abera Abdeta, Estifanos Tsige, Etsehiwot Adamu, Degefu Beyene, Semira Ebrahim, Elias Seyoum, Maritza Urrego, Tesfa Addis

## Abstract

**Background:** Considering the threat of antimicrobial resistance (AMR), Ethiopia implemented strategies to combat AMR, including partnering with the American Society for Microbiology (ASM) to conduct an AMR training program using the Project ECHO learning platform. ECHO AMR was used to virtually connect subject-matter experts with participating sentinel laboratories in remote locations to provide ongoing education, telementoring, and foster peer-to-peer learning and problem-solving in microbiology. In phase 1, the ASM had primary leadership in conducting sessions and project administration. In phase 2, roles and responsibilities transitioned from the ASM to the Ethiopian Public Health Laboratory (EPHI) with support from ECHO India. Here we describe the transition process and lessons learned. **Methods:** From December 2020–2021, biweekly 1-hour sessions were conducted for 8 sentinel laboratories. Each virtual session included a lecture led by a subject-matter expert, a case presentation by a participating laboratory, open discussion, and feedback via an end-of-session online survey. Following a transition plan, initial ASM-EPHI transition activities included formal administrative and logistical training, including participation in a 3-day Project ECHO-immersion program provided by ECHO India. Selected administrative and technical roles and responsibilities, including further developing their own SMEs, were transitioned from ASM to EPHI every 4 sessions. ASM conducted postsession reviews with EPHI and ECHO India to discuss successes and suggested improvements. **Results:** Leadership of ECHO AMR was fully transitioned to EPHI over 12 months. End-of-session surveys and postsession reviews indicated the transition process was successful, with EPHI staff leading the lectures, session coordination, and facilitation, and positive feedback from session participants. Challenges included variable sentinel site participation due to competing priorities such as COVID-19 testing and poor internet connectivity during the rainy season. Lessons learned included the need to use a gradual transition strategy with close monitoring, training facilitators to maintain implementation fidelity (level of reproducibility to conduct ECHO AMR as in phase 1) and improve participation, and assessing individual learning, using pretests and posttests. Recommendations included that ASM should remain as an external technical advisor to ensure program technical depth and session facilitators be trained to improve participation in the discussions. Implementation fidelity compared to phase 1 was considered moderate, with the gap primarily due to the need for dedicated release time from laboratory duties to ensure session leadership, coordination, and facilitation. **Conclusions:** Leadership and laboratory workforce capacity-building responsibility for AMR training was successfully transitioned from ASM to EPHI, promoting self-sufficiency in training and with far-reaching benefits in the global fight against AMR.

**Disclosures:** None

